# Infective Endocarditis in a Patient with Celiac Disease after Central Venous Catheter Insertion

**DOI:** 10.7759/cureus.1027

**Published:** 2017-02-13

**Authors:** Murtaza Sundhu, Suryanarayan Mohapatra, Salome Arobelidze, Parveen Gundelly, Anil Kumar Changarath Vijayan

**Affiliations:** 1 Fairview Hospital, Cleveland Clinic

**Keywords:** infective endocarditis, malnutrition, weight loss, celiac disease, central venous catheter, tricuspid valve

## Abstract

There is an increasing incidence of infective endocarditis secondary to central venous catheters, which is termed as 'healthcare-associated infective endocarditis'. There is an increased risk of getting infective endocarditis in conditions with malnutrition and also if the tip of the central venous catheter is deep in the right atrium close to the tricuspid valve.

We present a case of 31-year-old female who had all these risk factors. She was admitted to the hospital for the work up of the weight loss and was diagnosed with celiac disease. Central venous access was obtained because of poor peripheral intravenous access via the peripherally inserted central catheter which was complicated by thrombosis and removed after three days of insertion, and she was started on anticoagulation. Two weeks after being discharged, she presented to the emergency department with fever, shortness of breath, and had signs of congestive heart failure. A computed tomography of the chest for pulmonary embolism was taken and showed small clot burden pulmonary embolism and two cavitary lesions in the right lung. A transthoracic echocardiogram was taken and showed vegetation on the tricuspid valve and blood cultures were positive for Staphylococcus aureus. Hence, a diagnosis of infective endocarditis was made, and she was treated with intravenous antibiotics for a total of six weeks after a long and complicated hospital stay.

## Introduction

Infective endocarditis (IE) is a serious, life-threatening infectious syndrome. Healthcare-associated IE is becoming more common and the microbiologic epidemiology is changing as well [1-2]. The microbiological epidemiology of IE shows Staphylococcus aureus (*S. aureus*) is now the most common etiologic agent [1]. The emergence of *S. aureus* IE is partially due to increasing healthcare-associated IE [1, 3].

## Case presentation

A 31-year-old female with a past medical history of eczema and asthma was admitted to a tertiary care hospital for evaluation of significant weight loss and malnutrition. She was diagnosed with celiac disease based on the serological studies. A peripherally inserted central catheter (PICC) was inserted for poor intravenous access which was found to be close to the tricuspid valve area on confirmatory chest x-ray. The PICC was removed after three days as it was complicated by axillary vein thrombosis, and anticoagulation was started.

**Figure 1:**
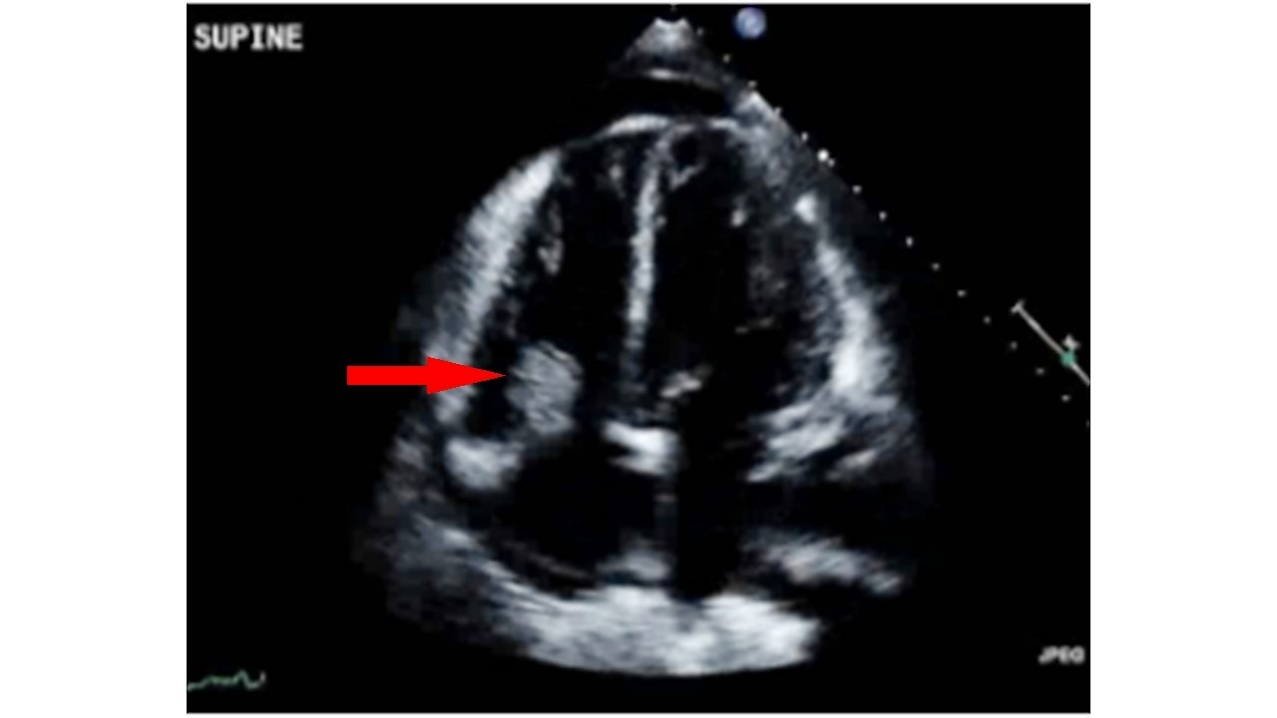
Transthoracic Echocardiogram A large mobile echodensity (2.65 x 1.52 cm) attached to the tricuspid valve leaflet that attaches to the RV free wall. It is homogeneous, globular, and moves freely between the RA and RV.

Two weeks after discharge, she presented to our emergency department due to fever, dyspnea, and leg swelling. Vital signs were: pulse of 112/min, blood pressure of 127/101 mmHg, respiratory rate of 47/min, temperature 97.5°F, and oxygen saturation was 100% on 4L oxygen. A physical examination revealed reduced air entry with crackles bilaterally on chest auscultation and no audible heart murmurs. Complete blood counts revealed white blood cell count of 8.10k/µL, hemoglobin of 7.8g/dL, and platelets of 95k/µL. The complete metabolic panel was within normal limits. NT ProBNP was >35,000pg/mL. A chest x-ray showed worsening left-sided pleural effusion and consolidations bilaterally. Computed tomography of the chest with contrast showed pulmonary embolism on the right side with small clot burden and cavitary right upper and right middle lobe pulmonary nodules. A transthoracic echo showed large mobile echodensity (2.65 cm x 1.52 cm) on the tricuspid valve with regurgitation (Figure 1).

The vegetation was attached to the tricuspid leaflet which was attached to the right ventricular free wall. The vegetation was globular, homogeneous, and moved freely between the right atrium and right ventricle. She was started empirically on intravenous vancomycin and meropenem. The patient went into respiratory distress requiring intubation. Blood cultures revealed methicillin-susceptible Staphylococcus aureus (MSSA), and the antibiotics were narrowed to oxacillin. She had a long and complicated hospital course. Her blood cultures remained positive for seven days despite appropriate antibiotics. A repeat echocardiogram showed a reduced size of the vegetation, and, therefore, surgery was not considered. She was given a total of six weeks of antibiotics. She followed up one and six months after discharge, and she was doing well. A repeat echocardiogram six months after discharge showed tricuspid valve with no vegetation and no regurgitation. The patient agreed to participate and was explained the nature and objectives of this study, and informed consent was formally obtained. No reference to the patient's identity was made at any stage during data analysis or in the report.

## Discussion

Infection of the heart valves (native or prosthetic), endocardial surface or intracardiac devices is known as infective endocarditis (IE) [4]. The incidence of IE in the United States has increased from 11 to 15 per 100,000 population from 2000 to 2011, respectively [5]. The diagnosis of infective endocarditis is made by the modified duke criteria [6].

There has been a shift in the epidemiology of IE, and more patients are having health care associated IE with *S. aureus* of the native valves rather than the subacute IE seen previously [1-3]. Most patients with IE have had chronic venous access or recent intravascular procedure in the last 60 days (60%), and morbidity and mortality associated with it remain high [1].

Central venous catheter insertion is one of the most notorious risk factors, and the potential mechanism is thought to be abrasion of the tricuspid leaflet and endocardium because of the close proximity to the tricuspid valve, and forceful and rapid jet of injections through the catheter [7]. Central venous catheters are more likely to cause IE when the tip of the catheter is deep, that is, in the right atrium [3]. IE also occurs more frequently in patients with burns [8], and burn patients lose proteins. Celiac disease is a protein-losing gastroenteropathy because of loss of villi in the small intestine. There are case reports of atopic eczema causing IE as well.

There have been studies to quantify the risk of infection between the central venous catheters and peripherally inserted central catheters, which showed the risk of infection to be the same [9]. The only difference between them is the time to development of infection, which is longer in the case of peripherally inserted central catheters [9].

Our patient had celiac disease, and a central venous catheter was placed which was deep in the right atrium as well. Hence, protein loss, central venous catheter, and direct irritation of the tricuspid leaflet all came into play in causing IE in our patient.

## Conclusions

Indications for and alternatives to central venous catheter insertion should be considered carefully in patients with malnutrition. Central venous catheter should only be used if absolutely necessary, and alternatives like ultrasound-guided peripheral intravenous access should be strongly considered before placing a central venous catheter.
